# Regression modelling of conditional morphogene expression links and quantifies the impact of growth rate, fitness and macromorphology with protein secretion in *Aspergillus niger*

**DOI:** 10.1186/s13068-023-02345-9

**Published:** 2023-06-02

**Authors:** Timothy C. Cairns, Tom de Kanter, Xiaomei Z. Zheng, Ping Zheng, Jibin Sun, Vera Meyer

**Affiliations:** 1 Chair of Applied and Molecular Microbiology, Institute of Biotechnology, Technische Universität Berlin, Straße Des 17. Juni 135, 10623 Berlin, Germany; 2 Tianjin Institute of Industrial Biotechnology, Chinese Academy of Sciences, Tianjin, 300308 People’s Republic of China; 3 Key Laboratory of Systems Microbial Biotechnology, Chinese Academy of Sciences, Tianjin, 300308 People’s Republic of China; 4 University of Chinese Academy of Sciences, Beijing, 100049 China; 5 College of Biotechnology, Tianjin University of Science & Technology, Tianjin, 300457 China

**Keywords:** *Aspergillus niger*, Macromorphology, Pellet, Cell wall, Chitin, Growth rate, Total protein, Tet-on, Genome editing, *dlpA*, *crzA*

## Abstract

**Background:**

Filamentous fungi are used as industrial cell factories to produce a diverse portfolio of proteins, organic acids, and secondary metabolites in submerged fermentation. Generating optimized strains for maximum product titres relies on a complex interplay of molecular, cellular, morphological, and macromorphological factors that are not yet fully understood.

**Results:**

In this study, we generate six conditional expression mutants in the protein producing ascomycete *Aspergillus niger* and use them as tools to reverse engineer factors which impact total secreted protein during submerged growth. By harnessing gene coexpression network data, we bioinformatically predicted six morphology and productivity associated ‘morphogenes’, and placed them under control of a conditional Tet-on gene switch using CRISPR-Cas genome editing. Strains were phenotypically screened on solid and liquid media following titration of morphogene expression, generating quantitative measurements of growth rate, filamentous morphology, response to various abiotic perturbations, Euclidean parameters of submerged macromorphologies, and total secreted protein. These data were built into a multiple linear regression model, which identified radial growth rate and fitness under heat stress as positively correlated with protein titres. In contrast, diameter of submerged pellets and cell wall integrity were negatively associated with productivity. Remarkably, our model predicts over 60% of variation in *A. niger* secreted protein titres is dependent on these four variables, suggesting that they play crucial roles in productivity and are high priority processes to be targeted in future engineering programs. Additionally, this study suggests *A. niger dlpA* and *crzA* genes are promising new leads for enhancing protein titres during fermentation.

**Conclusions:**

Taken together this study has identified several potential genetic leads for maximizing protein titres, delivered a suite of chassis strains with user controllable macromorphologies during pilot fermentation studies, and has quantified four crucial factors which impact secreted protein titres in *A. niger*.

**Supplementary Information:**

The online version contains supplementary material available at 10.1186/s13068-023-02345-9.

## Background

Fungi are used as microbial cell factories to produce a wide range of useful molecules, including foodstuffs, medicines, enzymes, and platform chemicals [1, 2]. These products can broadly be classified as proteins, enzymes, secondary metabolites, or organic acids, each of which represent industries worth several billion euros a year [3]. As fungi are able to utilize a wide range of nutrient sources and tolerate high concentrations of potentially toxic molecules, they are ideally suited to green biotechnological applications, whereby waste (e.g., from industry, agriculture or elsewhere) is converted into useful products. Indeed, applied fungal science has been identified as a key driver in the emerging bioeconomy [2] and is a major revenue stream for large biotech companies [4].

Filamentous fungi are distinguished from unicellular yeasts by their ability to produce tubular cells termed hyphae, which branch to form a mycelial network. Many important filamentous fungal cell factories are utilized in submerged fermentation, including *Aspergillus*, *Trichoderma*, *Penicillium*, *Thermothelomyces*, *Rhizopus*, and many others [5]. In liquid culture, filamentous fungi produce a range of macromorphologies, including approximately spherical pellets, irregular shaped clumps, dispersed mycelial fragments, or a combination of these growth types [6, 7]. Macromorphology is known to effect product titres and, additionally, rheological aspects of a fermenter. For example, protein titres may be highest when dispersed mycelial fragments are the predominant macromorphology [8, 9]. This is based on the hypothesis that this growth type contains a high number of actively extending hyphal tips, which is the subcellular location where many proteins are secreted into the growth media. One potential drawback of this growth type when compared to pellets is high medium viscosity, the build-up of micronutrient or oxygen gradients, difficulty in separating biomass from the desired product, and clogging of various bioreactor components [7]. Thus, optimizing strain macromorphology requires a balance between process engineering perspectives and optimal product titres [10].

While a large body of work has investigated how abiotic culture parameters impact macromorphology [11–16], targeted engineering by rationally modifying a gene of interest is relatively limited. Historically, strain engineering has predominantly used iterative rounds of mutagenesis and phenotypic screening [3]. While certainly effective in generating a portfolio of optimized industrial lineages (some of which have modified macromorphologies [17]), in many instances the underlying mechanisms which result in a hyperproduction phenotype or improved strain process engineering performance are unknown [18]. Such mechanistic explanations could conceivably occur at multiple conceptual levels, including molecular (e.g., by increasing the efficiency of a key biosynthetic enzyme or transporter [19]), metabolic (e.g., through enhancing flux of a crucial pathway [20]), subcellular (e.g., by improving delivery of biosynthetic enzymes to their site of action [21]), morphological (e.g., by elevating branching rates to produce high levels of actively secreting tips [22]) or macromorphological (e.g., by reducing pellet diameter to improve diffusion of oxygen or nutrients [23]).

Rational design and control of optimal hyperproducing strains will require integrated understanding of how these complex factors are interconnected. In a proof of principle study, we previously assessed how filamentous hyphal morphology impacts macromorphological development in the organic acid and protein producing fungus *Aspergillus niger* [24]. By quantifying filamentous growth and submerged macromorphology of conditional expression mutants, it was possible to use multiple linear regression modelling to predict that changes in strain hyphal tip number, but not length, did indeed cause measurable impacts in macromorphological development. Crucially, this approach analysed filamentous growth and macromorphology across multiple genetic backgrounds, which enables greater confidence when compared to similar experiments using a single isolate [24].

An additional challenge for morphology engineering at the genetic level is identifying high priority candidates for functional analysis from over 14,600 predicted genes in the *A. niger* genome [25]. We have previously generated new predictions of gene function at a near-genome level in *A. niger* using coexpression networks [26]. This approach is predicated on the hypothesis that genes which are robustly coexpressed over hundreds of conditions are likely to function in similar processes. By defining genes which are highly coexpressed with the citric acid synthase encoding gene *citA*, we identified 57 high priority candidates for strain engineering as the tricarboxylic acid cycle is crucial for (i) organic acid production and (ii) generating amino acid precursors for protein synthesis. A final refinement of these coexpression data was to identify genes with a putative role in morphology, thus generating a list of *A. niger* ‘morphogenes’ that may have industrially relevant morphological impacts [24].

The aim of this study was to further develop this approach by (i) generating conditional mutants in *A. niger*, (ii) conducting quantitative phenotypic screens, and (iii) use multiple linear regression modelling to predict which factors impact total secreted protein during submerged culture. Genome editing was used to place six predicted morphogenes under control of a Tet-on conditional expression cassette, after which gene expression was titrated and filamentous growth, submerged macromorphology, and total protein titres quantified. In order to estimate how other consequences of controlled gene expression impacted productivity, we conducted phenotypic screens under various abiotic perturbations. This enabled us to predict, for example, that strain cell wall integrity impacts total secreted protein.

## Results

### Constructing *A. niger* morphogene expression mutants using the Tet-on conditional expression cassette

In this study, we selected six morphogenes from a total of 57 [24] (Table 1) and generated conditional expression mutants by placing them under control of the synthetic Tet-on cassette as previously described [21, 27]. The 5′ UTR of each gene of interest (Table 1) was screened for sgRNA sites and cut using a Cas9 nuclease [21, 27, 28]. In order to place the titratable cassette immediately upstream of the gene of interest, we introduced 40 bp targeting sequences on the 5′ and 3′ regions of the Tet-on cassette by PCR, which was co-transformed with sgRNA (Additional file 1: Table S1). Primary transformants were purified twice on agar supplemented with hygromycin, after which presence of the Tet-on cassette immediately upstream of the gene of interest was confirmed by PCR (data not shown). A second quality control step utilized Southern blotting, which confirmed the cassette was indeed at the intended locus, and furthermore, had integrated into the recipient genome as a single copy (Additional file 1: Table S2 and Fig. S1). Strains which passed both steps are described in Table 1.

**Table 1:** Strains generated in this study

Gene name^a^	Gene	Strain name (this study)	Predicted functional category^b^	Function or experimental characterization as described in yeast and filamentous fungi^b^	Evidence of morphogene function
*crzA*	An01g13700	TK26.2	Transcription factor	Transcription factor which activates expression of stress response genes; nuclear localization is positively regulated by calcineurin-mediated dephosphorylation	[29]
*amgA*	An12g10750	TK28.5	Transcription factor	Gene deletion impacts shmoo formation in *Schizosaccharomyces pombe*	[30]
*rscI*	An11g02670	TK38.5	Component of the RSC chromatin remodelling complex	Rsc9 is involved in both repression and activation of mRNAs regulated by target of rapamycin (TOR)	[31]
*strA*	An16g01520	TK39.1	WD-repeat protein that regulates sexual development	Regulates sexual development and localizes to endoplasmic reticulum in *Aspergillus nidulans*	[32]
*cihA*	An07g01060	TK51.1	Has domains with predicted role in cell wall macromolecule catabolic process	Possible cell wall associated protein	[33]
*dlpA*	An08g10160	TK53.1	Dehydrin-like protein	Deletion mutant impacts conidiation in *A. nidulans*	[34]

^a^Naming follows the nomenclature for filamentous fungi

^b^Predicted functional categories were derived from the Ensemble or FungiDB databases

### Impact of morphogene expression on *A. niger* growth during solid cultivation

**Fig. 1:**
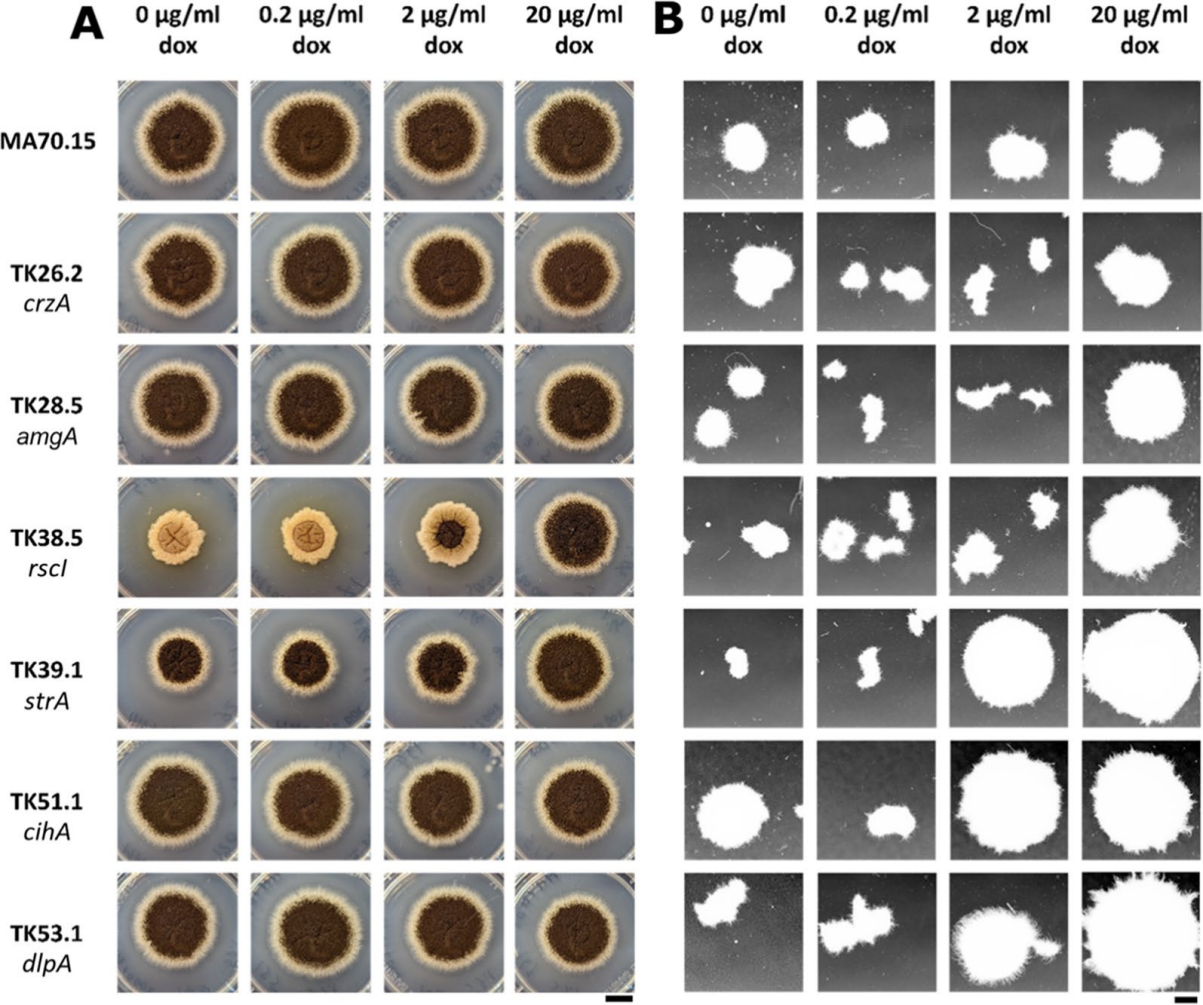
Growth of the morphogene mutants and MA70.15 control on solid and liquid media supplemented with various Dox concentrations. Representative images of radial growth after 5 days incubation at 30 °C on MM agar (left panels). Scale bar = 1 cm. Representative images of submerged growth in MM for 72 h at 220 RPM, 30 °C (right panels). Scale bar = 1 mm

**Fig. 2:**
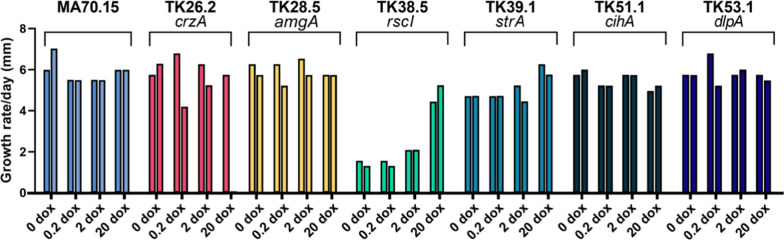
Radial growth rates of the morphogene mutants and MA70.15 control at different Dox concentrations on solid MM. The growth rate per day was calculated by measuring the radial growth of the *A. niger* colonies between 72 and 120 h. Duplicate replicates are depicted

In order to determine impacts of morphogene expression on colony growth and development, we quantified radial growth on solid minimal media (MM) supplemented with 0, 0.2, 2 and 20 µg/ml Dox (Figs. 1 and 2). These Dox concentrations, respectively, model null, minor, moderate, and overexpression throughout this study as previously shown [9, 20, 21, 24, 27, 35, 36]. This demonstrated a clear role of gene *rscI* in *A. niger* growth and development, with colonies for mutant TK38.5 displaying severe growth reduction under 0, 0.2, 2 µg/ml Dox, which was partially restored by supplementing media with 20 µg/ml Dox. Colonies were aconidial (0, 0.2 µg/ml Dox) or displayed a defective conidial phenotype (2 µg/ml Dox) which could only be rescued by expressing *rscI* at the highest level used in this study. Mutant TK39.1 demonstrated reduced radial growth rates under 0, 0.2, 2 µg/ml Dox, which were comparable to the progenitor control using 20 µg/ml Dox, clearly indicating a role of *strA* in colony growth. However, in contrast to isolate TK38.5, no defect in conidiation was observed in TK39.1. Thus, impacts of gene expression on *A. niger* colonies were detected in two of the six strains in this study.

**Fig. 3:**
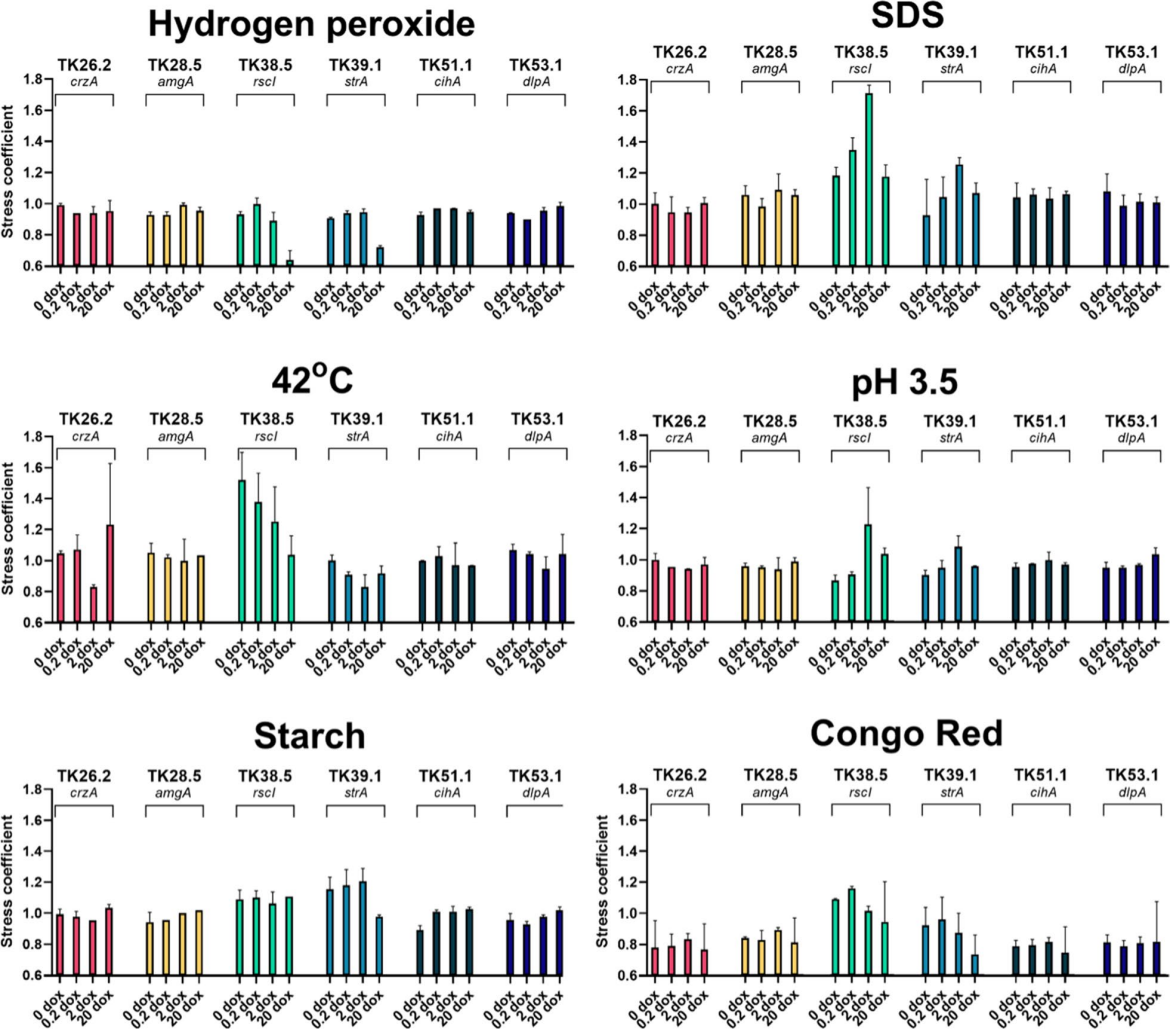
Growth coefficients of the morphogene mutants following various abiotic perturbations. Elevated and reduced radial growth of the mutant under stress relative to MM control are reported as > 1 and < 1, respectively. See “Methods” section for full details

We further quantified radial growth of the mutants under numerous abiotic stress conditions to further determine the impacts of titratable morphogene expression [37]. We perturbed protein stability and folding using sublethal oxidative stress or sodium dodecyl sulphate (Fig. 3) [37]. In a proxy measurement of general secretion capacity, strains were also screened on media where glucose was replaced with starch as the sole carbon source, conditions which require extracellular starch degrading enzymes for growth. Fitness under cell wall stress was probed using Congo red, a dye that binds chitin [38]. We also measured growth under decreased pH (3.5) and fitness under heat stress (42 °C). Data are reported as growth coefficients, whereby the magnitude of change between mutant and progenitor control strain is compared between standard minimal media and stress conditions, so that elevated and reduced radial growth of the mutant under stress is reported as > 1 and < 1, respectively [37]. We detected both elevated and decreased growth coefficients at specific Dox concentrations for a variety of strains (Fig. 3). This included, for example, titratable growth coefficients for mutant TK38.5 on elevated temperature, demonstrating that *A. niger* grew more efficiently at high temperatures when *rscI* expression was low. Interestingly, both mutants TK38.5 and TK39.1 demonstrated sensitivity to hydrogen peroxide when media were supplemented with 20 µg/ml Dox, indicating high levels of *rscI* and *strA* impact *A. niger* responses to oxidative perturbation. Most strains demonstrated growth coefficients < 1 when grown on sublethal concentrations of Congo red. This suggests general sensitivity of the mutants to cell wall stress, with the exception of mutants TK38.5 (all Dox concentrations) and TK39.1 (0, 0.2 and 2 µg/ml Dox). Indeed, with the exception of growth on cell wall stress, mutants TK26.2, TK28.5, TK51.1, and TK53.1 performed comparably to the control in standard minimal media and stress conditions. Notably, we did not detect any major defects in any mutant/Dox concentration when growth on starch media, suggesting all strains are able to secrete starch degrading enzymes to maintain growth rates.

### Impact of morphogene expression on *A. niger* hyphal growth

**Fig. 4:**
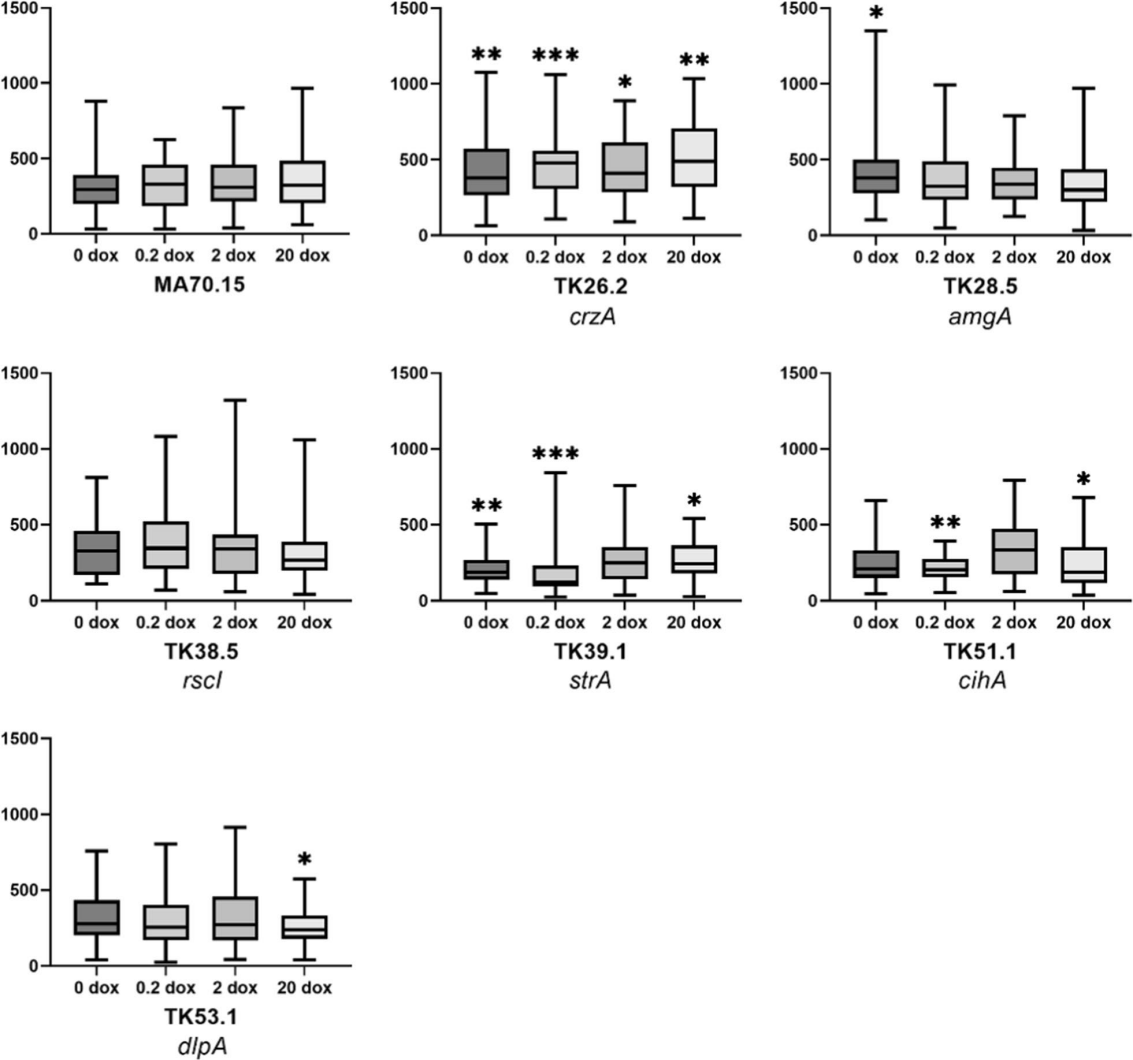
Box plot representation of hyphal length (µm, *y*-axis) following incubation on solid MM at 30 °C for 18 h. Morphogene expression was titrated using the four described Dox concentrations. Asterisks indicate where hyphal length of mutant isolate significantly deviates from MA70.15 control at the respective Dox concentration (Students *t*-test *p < 0.05, **p < 0.01, ***p < 0.001). Approximately 30 hyphae per strain/Dox condition were quantified. *Y*-axis: hyphal length (µm)

We have previously demonstrated that hyphal tip number, but not length, impacts *A. niger* macromorphology during shake flask cultivation and citric acid production [24]. We therefore quantified how titration of morphogene expression in the mutants affected hyphal length (µm, Fig. 4) and tip number, (Additional file 1: Fig. S2) following 18 h growth on minimal agar. From these data we also calculated growth unit as a measure of branching frequency (hyphal length/tip number, Additional file 1: Fig. S3). This analysis identified multiple impacts of morphogene expression levels on *A. niger* hyphal morphology. For example, mutant TK39.1 (in which *strA* is under Tet-on control) demonstrated a hyperbranching phenotype relative to progenitor control at all tested Dox concentrations. This observation was partially titratable, with clear increase in hyphal growth unit in TK39.1 when 20 µg/ml Dox was supplemented to growth media, thus demonstrating *strA* impacts branching. Similarly, a reduction in hyphal growth unit for strain TK53.1 relative to control was observed when grown on 2 or 20 µg/ml Dox, indicating that expression of *dlpA* also impacts branching frequency. Interestingly, isolate TK26.2 demonstrated significant increase in both tip number and hyphal length at all analysed Dox concentrations. These data indicate that titration of *crzA* expression causes a detectable increase in hyphal growth rate, but does not impact branching frequency. Additionally, expression of *cihA* in mutant TK51.1 caused a reduction hyphal length and tip number compared to MA70.15 control under 0, 0.2 and 20 µg/ml Dox. While these differences were only statistically significant for 0.2 µg/ml Dox, a general trend suggesting minor yet detectable reduction in hyphal growth rate was observed in this mutant under these concentrations. Moreover, a slight reduction was observed in hyphal growth units when mutant TK51.1 was grown on 20 µg/ml Dox (*p* < 0.05). Thus, we conclude that expression of *cihA* has various impacts on hyphal morphology in *A. niger*, including reduced growth rates (0, 0.2 and 20 µg/ml Dox) and hyperbranching (20 µg/ml Dox), yet grows comparably to the progenitor control when this gene is expressed using 2 µg/ml Dox. It is notable that hyphal morphology was comparable between TK38.5 and control at all Dox concentrations given the strong defects in colony growth and conidiation in this mutant (Fig. 1A). Thus, strong modulation in colony development in this mutant is not due to defects in early hyphal growth, suggesting that gene *rscI* impacts *A. niger* after 18 h growth on solid agar. In summary, phenotypic quantification at both colony and hyphal level identify various and titratable impacts of morphogene expression during *A. niger* growth on solid agar.

### Impact of morphogene expression on *A. niger* macromorphology during submerged cultivation

**Fig. 5:**
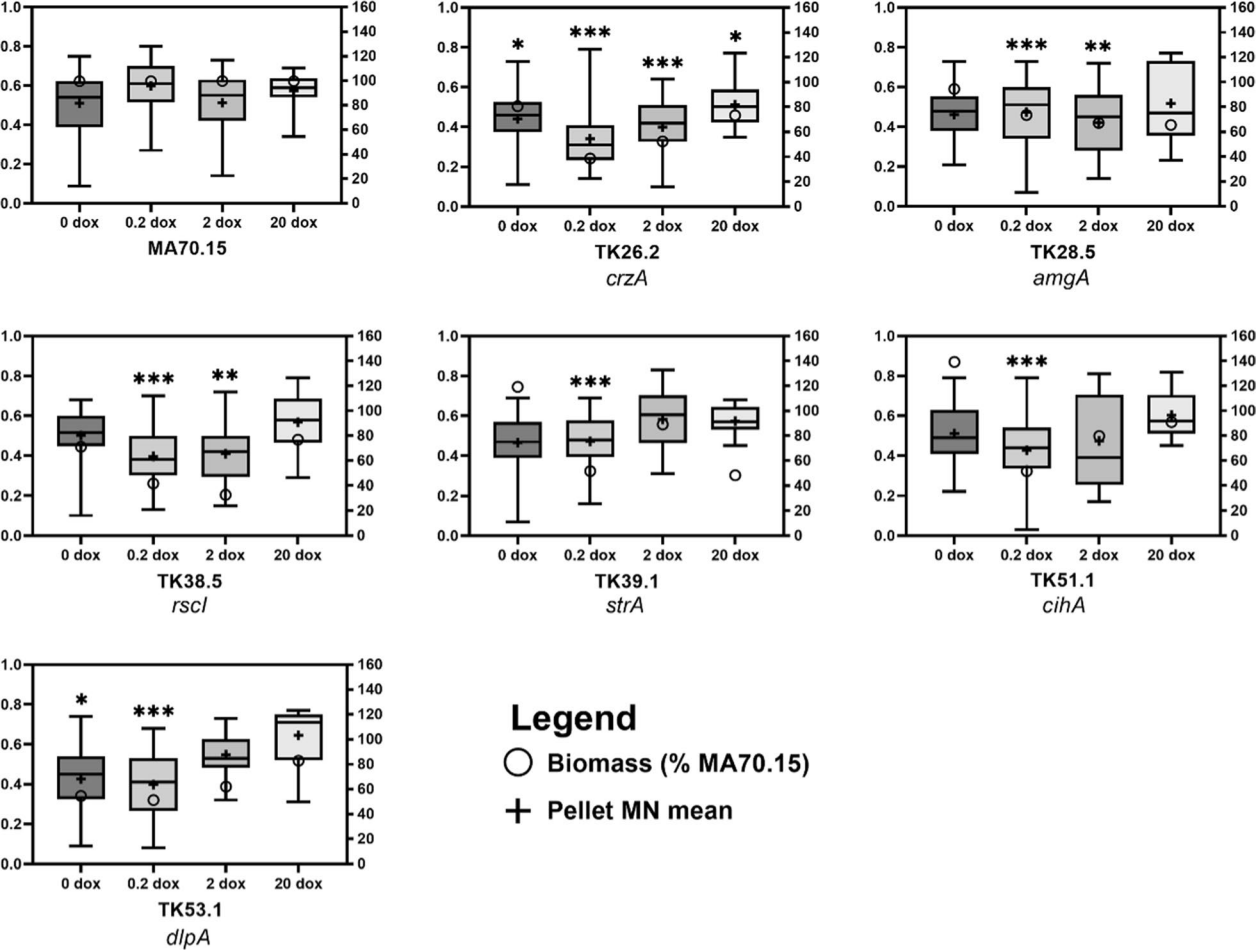
Quantification of submerged culture biomass and pellet MNs following titration of morphogene expression. *Y*-axis: pellet MNs, which vary between 0 and 1 and are represented by boxplots. + Indicates mean MN values, and the middle horizontal line indicates the median. Asterisks indicate where MN of mutant isolate significantly deviates from MA70.15 control at the respective Dox concentration (*t*-test, *p < 0.05, **p < 0.01, ***p < 0.001). Values are from triplicate biological replicates. Right axis: ○ indicates biomass given as a percent of MA70.15 control at the respective Dox concentration

**Fig. 6:**
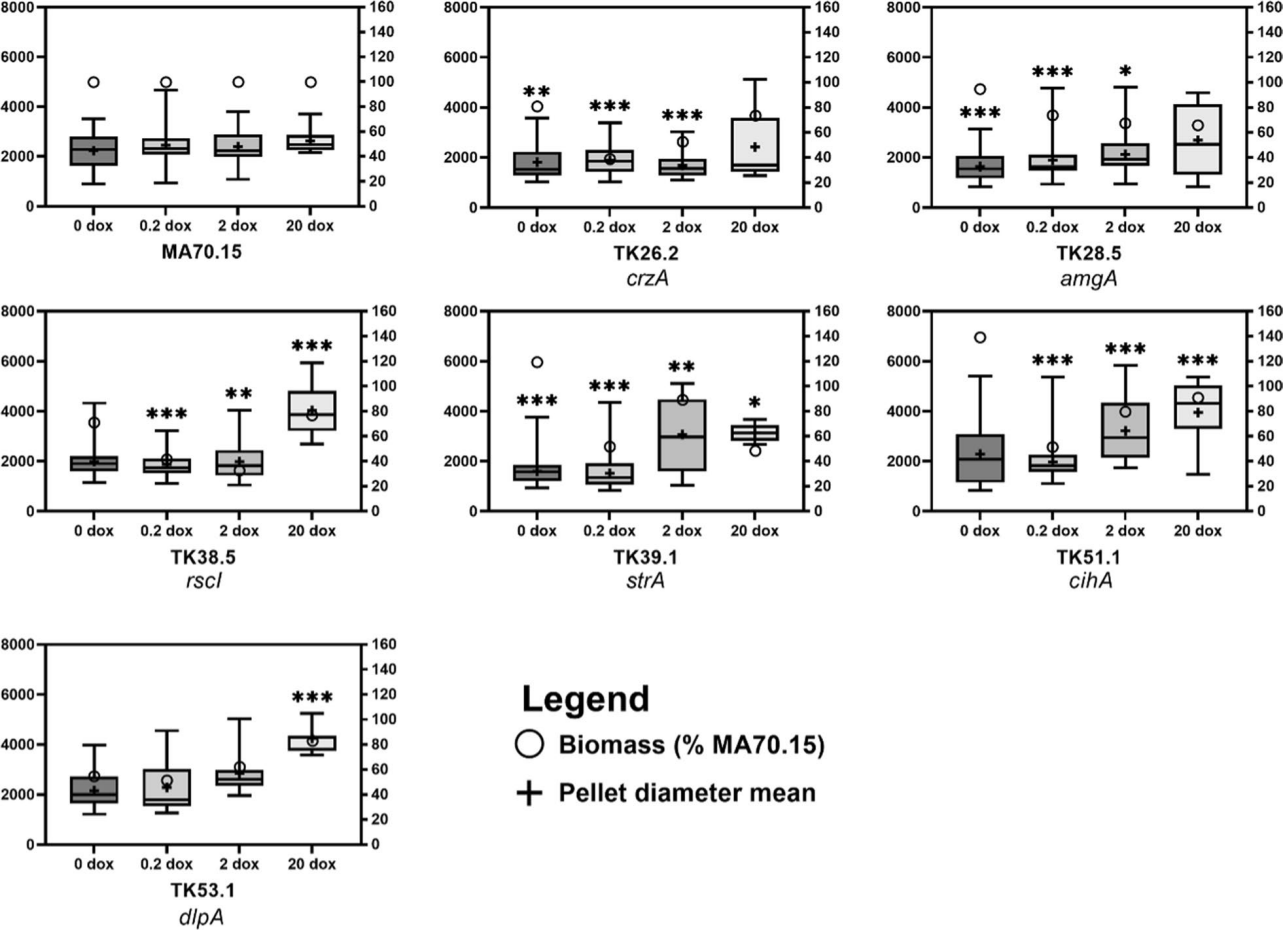
Quantification of submerged culture biomass and pellet diameter following titration of morphogene expression. *Y*-axis: pellet maximum diameter (µm) is represented by boxplots. + Indicates mean pellet diameter, and the middle horizontal line indicates the median. Right axis: biomass is given as a percent of MA70.15 control at the respective Dox concentration. Asterisks indicate where diameter of mutant isolate significantly deviates from MA70.15 control at the respective Dox concentration (*t*-test, *p < 0.05, **p < 0.01, ***p < 0.001). Values are from triplicate biological replicates

To test whether morphogene expression impacted submerged macromorphologies, we quantified growth in shake flask models of protein fermentation (Figs. 1, 5 and 6). The automated image analysis plugin MPD calculates various pellet Euclidean parameters, including area, diameter, solidity, and aspect ratio which are used to generate a morphology number (MN, [27]). These values vary between 1 (a perfect circle) and 0 (a hypothetical one-dimensional line) and are useful to interpret macromorphological screens [39]. This analysis identified distinct macromorphologies amongst the mutants following titration of morphogene expression (Fig. 5).

When interrogating specific Euclidean parameters, it was clear that pellet diameter was modulated in the conditional expression mutants. Diameter was reduced in strains TK26.2 and TK28.5 when grown in media supplemented with 0, 0.2 or 2 µg/ml Dox, yet was comparable to control when grown under 20 µg/ml Dox (Fig. 6). Isolate TK53.1 had comparable pellet diameter relative to the control except when expressed using 20 µg/ml Dox, where pellet diameter increased. Interestingly, mutant TK38.5, TK39.1 and TK51.1 displayed both significant reduction in pellet diameter at lower Dox concentrations, and increased diameter relative to control at 20 µg/ml Dox. Similar changes observed for the morphogene mutants for pellet aspect ratio and surface solidity (Additional file 1: Figs. S4 and S5), demonstrating that all six morphogenes analysed in this study do indeed impact *A. niger* macromorphology during submerged cultivation.

**Fig. 7:**
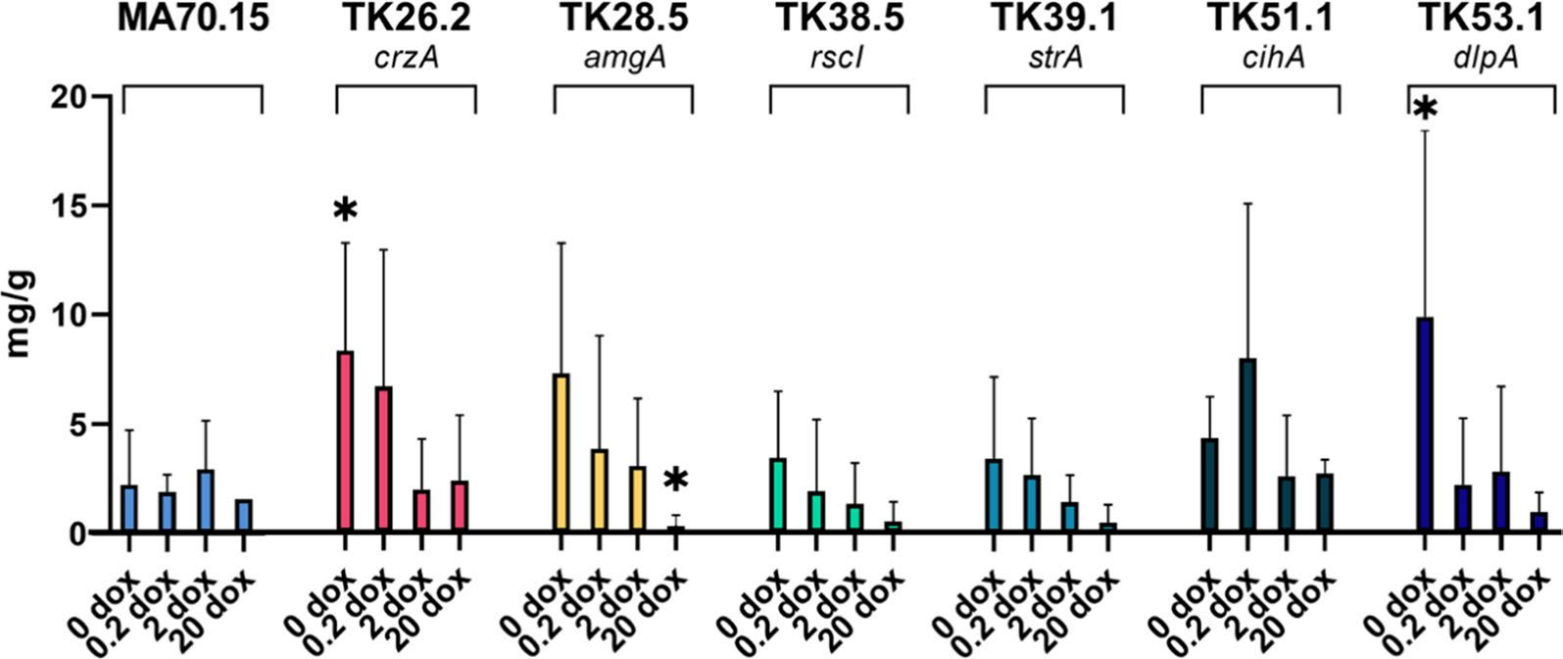
Secreted protein titres in submerged cultivation. Total secreted protein (mg) was determined from culture supernatants using a Bradford assay, which was normalized to fungal biomass (g dry weight). Error bars report standard deviation from triplicate biological replicate. *p* values < 0.05 are depicted with an asterisk. Asterisks indicate where protein titres of mutant isolate significantly deviates from MA70.15 control at the respective Dox concentration (*t*-test)

We also measured total secreted protein for each strain/Dox concentration which was normalized to biomass, identifying significantly elevated (TK26.2 and TK53.1, 0 µg/ml Dox), and reduced protein titres (TK28.5, 20 µg/ml Dox) relative to MA70.15 controls (p < 0.05, Fig. 7). These data suggest that *crzA* and *dlpA* might be promising leads for optimizing protein fermentation in *A. niger*.

### Multiple linear regression analysis to identify factors that impact secreted protein titres

In order to identify which, if any, aspects of strain performance impact titres of total secreted protein, we performed regression analysis. Radial growth rates (Fig. 2), growth coefficients on sublethal stress (Fig. 3), hyphal length following germination (Fig. 4), and Euclidean pellet parameters during submerged growth (Fig. 6 and Additional file 1: Figs. S4 and S5) were defined as independent variables, constituting a dataset of 308 quantitative phenotypic data. Total secreted protein (mg/g) was defined as the dependent variable. This analysis identified four factors which statistically impact protein titres; including radial growth rate (*p* < 0.01), pellet diameter (*p* < 0.001), fitness under cell wall stress (*p* < 0.001), and fitness under elevated temperature (*p* < 0.01, Table 2). Radial growth rate and fitness under heat stress were positively correlated with protein titres, whereas diameter of submerged pellets and fitness under cell wall stress were negatively associated with productivity (Table 2).

**Table 2:** Prediction of protein titres using regression modelling

	*P*-value	Coefficient	Change relative to MA70.15 control strain (%)	Predicted protein concentration (mg/g)
			0	2.83
Growth rate	0.0041	0.6592	− 10	1.97
			− 20	1.12
			− 30	0.48
Fitness under heat stress	0.0047	10.6824	− 10	2.62
			− 20	1.55
			− 30	0.48
Fitness under cell wall stress	0.0009	− 13.4059	− 10	5.03
			− 20	6.37
			− 30	7.71
Pellet diameter	0.0002	− 0.0020	− 10	4.14
			− 20	4.60
			− 30	5.05

Test variables were only incorporated into the regression model where *p* < 0.05. Hypothetical decreases (10, 20, 30%) in the indicated strain parameter relative to the MA70.15 control were used to calculate predicted protein titres (mg/g)

**Fig. 8:**
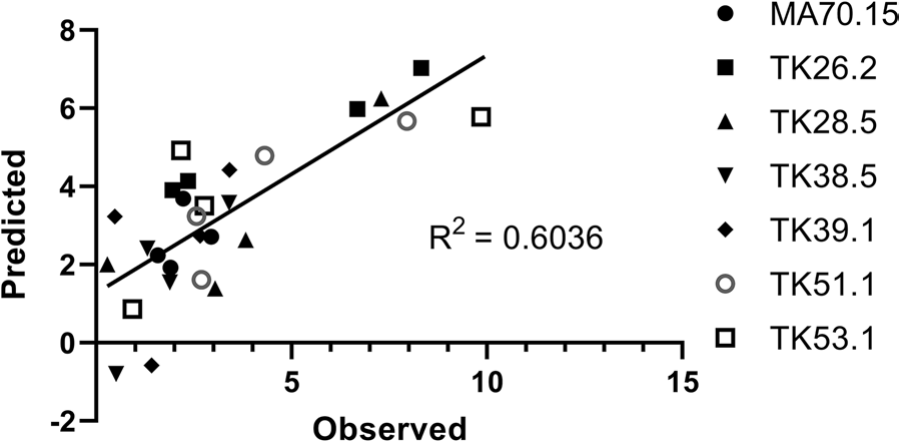
Correlation of observed and predicted secreted protein titres in submerged culture. Four parameters indicated in Table 2 were used to predict protein titres (mg/g) for each respective strain/Dox concentration. These were plotted against those observed experimentally (Fig. 7)

The regression analysis also enabled us to quantify how changes in these four parameters impact protein titres. For example, a 10% reduction in radial growth rate, or fitness under heat stress, is predicted to cause total secreted protein during submerged growth to drop to ~ 69% and ~ 93% of the MA70.15 control, respectively. In contrast, a 10% reduction in strain pellet diameter, or fitness under cell wall stress, is predicted to cause protein titres to increase to 146% or 178% of the control, respectively. Plotting total secreted protein titres predicted by the regression model against those observed in shake flask culture revealed reasonable concordance (*R*^2^ = 0.60, Fig. 8), indicating that 60% of variation in total secreted protein titres can be attributed to modifications in these four parameters.

**Fig. 9:**
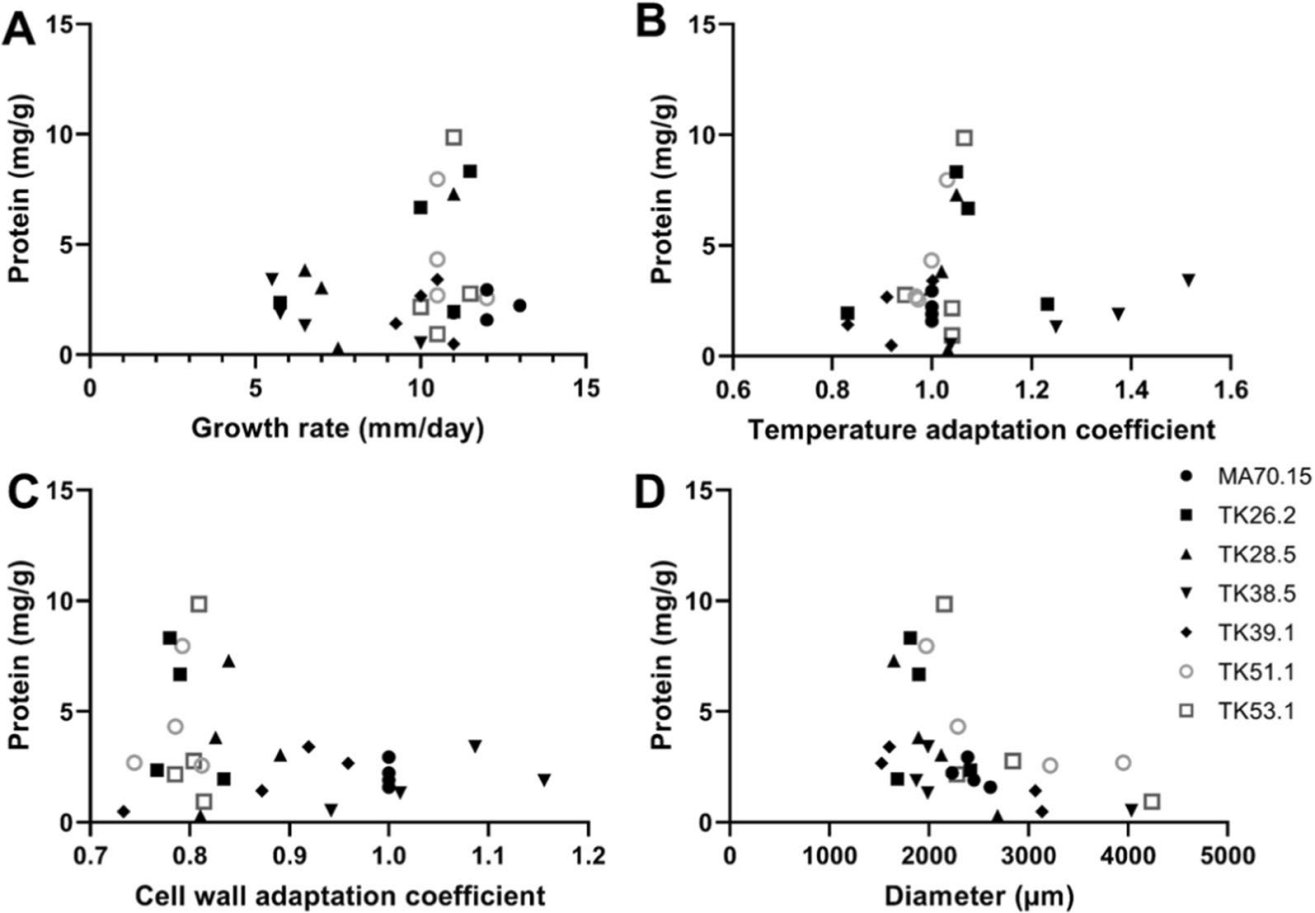
Pairwise correlations between total secreted protein titres and four key strain parameters. Data points correlate protein with the indicated parameter for a specific strain/Dox concentration

Finally, plotting the respective four parameters against total secreted protein allowed refinement of the main conclusions from the regression analysis. Specifically, elevated product titres tended to be observed in strains with the following: (i) a robust growth rate of ~ 10 mm/day (Fig. 9A); (ii) a growth coefficient at elevated temperature of 1-1.2; (iii) a growth coefficient on cell wall perturbation of ~ 0.8 (Fig. 9C); and (iv) pellet diameter of ~ 2000 µm (Fig. 9D).

## Discussion

Fungal cell factories are major producers of a wide range of useful molecules [2]. However, strategies for engineering the next generation of hyperproducing industrial strain lineages are currently limited as the phenotypic parameters that cause elevated titres of a desired molecule are not comprehensively understood [10]. Moreover, pleiotropic and multifactorial consequences of gene functional studies make identifying key factors that enhance productivity challenging. In this study, we have used the multipurpose cell factory *A. niger* to interrogate how total protein titres are connected to quantitative strain phenotypes, ranging from growth rate, response to stress, and submerged macromorphology.

From a tool development perspective, this manuscript has generated six isolates with controllable macromorphologies during shake flask culture. Despite the inherent heterogeneity of macromorphology that is observed during submerged culture of filamentous fungi [7, 40–42], this study demonstrated that pellet parameters of the described conditional mutants can be modified by addition of Dox, including diameter, aspect ratio, surface structure and morphology number. Given that all conditional expression mutants were constructed in the *ku70*^*−*^ and *pyrG*^−^ isolate MA70.15 [43], it will be easy for interested users to harness the strain collection as a chassis expression resource. Specifically, targeting exogenous DNA cassettes to a desired locus is simple due to *ku70* disruption. Additionally, the highly utilized *pyrG* recyclable marker [3], and over-expression locus [26], remain available in all the strains. It should be noted that polycistronic gene expression systems have been developed in *A. niger* [44]*.* When combined with the mutants and facile molecular tools described in this manuscript, it will be possible to test if macromorphological changes can be engineered to elevate titres of any protein, organic acid, or secondary metabolite products of interest in *A. niger*.

By analysing quantitative measurements of strain phenotypes using regression modelling, we were able to show that total secreted protein is impacted by radial growth rate, pellet diameter, and fitness under elevated temperature or chitin-based cell wall perturbation. These data for the first time quantify several aspects of *A. niger* biology, most obviously that *A. niger* protein secretion is coupled to growth by trafficking of cargo-loaded vesicles to the extending hyphal tip [45, 46]. Our data demonstrate that defective secretion in liquid culture can be predicted by defective radial growth rates on solid agar (Table 2). The regression model is consistent with the well-established role of the fungal cell wall in protein secretion [47]. Congo red binds and disrupts chitin assembly in fungal cell walls [38], and our data suggest that increased sensitivity to this abiotic stress is correlated with elevated secreted protein during liquid culture. We propose that this can be mechanistically explained by enhanced leakage of protein molecules across weakened cell wall in mutants that are sensitive to chitin stress. This hypothesis is supported from other studies, where RNAi knockdown of chitin synthesizing genes in *A. niger* and *Penicillium chrysogenum* resulted in elevated citric acid (40%) and penicillin product titres (~ 30–40%), respectively [48, 49]. Our data support growing evidence that genetic targeting of chitin synthases may be an effective method to elevate product titres in *A. niger* and other fungi.

Our analysis also suggested that pellet diameter plays a crucial role during fermentation, with protein hyperproducing strains having small pellets (Table 2). Other studies have shown similar trends, for example, an increase in total secreted protein following decreased pellet diameter in conditional *aplD A. niger* mutants [27] or reduction of pellet size by addition of insoluble particles to growth media [50]. These data are consistent with growing evidence that a limiting step in productivity is diffusion of nutrients and oxygen into the pellet core [10, 23], and suggest that small pellets perform favourably when compared to larger ones.

The regression model also demonstrated that hyphal tip number or branch rate was not predicted to impact protein titres. Previously, a hyperbranching phenotype was found to be insufficient to elevate secretion of the industrially relevant glucoamylase enzyme in *A. niger* [51]. However, GlaA titres could be increased to 400% of the progenitor control by overexpression of the *glaA* gene in a hyperbranching chassis strain [51]. Thus, our data are consistent with the hypothesis that hyperbranching alone is not sufficient to impact protein titres in *A. niger*, and that other modifications are necessary to push desired proteins through the secretory system.

A useful aspect of the regression analysis applied in this study is that it can be used to assess how observed protein titres differ from those predicted by the model (Fig. 8). This approach suggested that ~ 60% of the variation in protein titres was indeed due to the four factors discussed above. Firstly, it was remarkable that such large differences in strain productivity can be predicted based on only four strain phenotypes. Secondly, quantitative predictions of the magnitude for how these factors impact protein titres may enable the prioritization of key characteristics for strain engineering programs, with our analysis suggesting isolates with (i) chitin-defective cell walls; (ii) small pellets, (iii) robust growth at moderate and high temperatures are high priority strains.

This study has also demonstrated that mining coexpression networks is an unbiased approach that identifies high priority genetic leads to increase protein titres during fermentation. In future, this strategy can be expanded to other product classes (e.g., organic acids and secondary metabolites). Specifically, we demonstrate that reduced expression of genes predicted to encode the transcription factor *crzA* and a putative dehydrin *dlpA* elevate total protein in shake flask supernatant. These data need scale-up and further verification, including testing the respective conditional expression strains in controlled bioreactor cultivations, and validation of whether elevated total protein correlate with increased titres of useful molecules, such as the glucoamylase GlaA. Mechanistic explanations for how *crzA* and *dlpA* impact any of the four key factors identified from the regression model, or indeed other aspects of protein synthesis or secretion, are beyond the scope of this study. It is interesting to note, however, that in yeast the calcium regulated transcription factor Crz1 regulates the fortification of the cell wall with chitin in response to perturbation with a the small molecular weight antifungal protein (AFP) [52]. Possibly, reduced expression of the *crzA* gene in *A. niger* may modulate cell wall chitin content, thus elevating secreted protein titres, a hypothesis we will test in future studies. Elevated protein titres in *crzA* and *dlpA* conditional expression mutants are at least consistent with the coexpression of these genes with the citric acid synthase *citA* (Table 1). It is also possible that modifying these genes could somehow impact the citric acid cycle to maximize production of protein precursors, although this is highly speculative at this point.

## Conclusion

*A.niger* is a multipurpose cell factory used to produce a diverse portfolio of molecules during submerged fermentation. The molecular, cellular, morphological, and macromorphological factors that result in optimal strain performance are not yet fully understood. We have generated six conditional expression mutants and used these to reverse engineer four factors which impact total secreted protein. Using regression modelling, we predict these factors account for 60% of variation in secreted protein titres during shake flask cultivation, suggesting that they are priority processes to be targeted in engineering programs. This study has also identified *crzA* and *dlpA* as potential genetic leads for maximizing protein titres in *A. niger*. We believe that the fundamental insights and tools generated in this study can guide morphology engineering in *A. niger* and other filamentous fungal cell factories.

## Methods

### Materials and methods

#### Microbial strains

Fungal strains used in this study are given in Table 1. MA70.15 (*pyrG*^−^) was used as progenitor isolate [43]. All bacterial plasmids were propagated in *Escherichia coli* DH5α using 100 µg/ml ampicillin as selection. Plasmids are given in Additional file 1: Table S3.

#### Coexpression analysis and identification of morphogenes

Morphogenes were taken from a previous analysis [24], whereby genes robustly coexpressed (Spearman correlation coefficient > 0.7) with a citric acid synthase encoding gene *citA* (An09g06680) across 283 microarray experiments were shortlisted. Genes were analysed by literature searches to identify those with a predicted role in *A. niger* growth or morphology as previously described [24].

#### Media and culture conditions

Strains of *A. niger* were grown on at either solid or liquid minimal medium (MM) [9]. Hygromycin was only added during purification of transformants on solid agar. Shake flask models of fermentation were conducted at 220 RPM, 30 °C, for 72 h, with three independent replicates conducted per strain/Dox concentration. All agar plates and liquid cultures had a final concentration of 4 mM uridine. For calculation of growth coefficients under stress, sublethal concentrations of respective compound were determined through cultivation of MA70.15 on individual MM plates containing a dilution series of the stress factors as described in [37]. Mutant and progenitor strains were grown on MM plates or MM plates supplemented with the indicated compound (200 µg/ml Congo Red, 2 mM H_2_O_2_, 0.005% SDS). For growth at lower pH, MM agar was adjusted using 1 M HCl. Glucose was replaced with starch to a final concentration of 1% where indicated.

#### Molecular techniques

All molecular techniques were performed according to standard procedures described previously [9]. *A. niger* transformation and genomic DNA extraction were performed as described elsewhere [53], with 5–10 µg/ml doxycycline (Dox) added to primary transformation plates and sub-culture media.

CRISPR-mediated genome editing was conducted as described previously [27]. All plasmid sequences will be made available on reasonable request. 2 µg of the Cas9 encoding plasmid Cas9-Hyg [Zheng et al., in preparation] was co-transformed with 2 µg purified sgRNA and 2 µg donor constructs into *A. niger* MA70.15 protoplasts as previously described [28]. Following selection (200 μg/ml hygromycin and 5–10 μg/ml Dox) and duplicate purification (200 μg/ml hygromycin and 5–10 μg/ml Dox) on MM supplemented, genomic DNA was extracted from putative transformants. Insertion of the donor cassette at the respective promoter region was confirmed by diagnostic PCR using verification primers. Isolates generated in this study were confirmed for single integration of the Tet-on cassette at the target locus using Southern blot probe and the *fraA* promoter of the Tet-on cassette [36].

#### Growth quantification on solid media

Hyphal growth was measured on MM agar slices that were sufficiently thin (approx. 1 mm) for light microscopic analysis as described previously [27]. Briefly, 10 µl of 1 × 10^4^ spores/ml of mutant or control isolates were spotted in duplicate onto the agar slice, air dried, and incubated at 30 °C for 18 h after which images of fungal growth were captured using a Zeiss Axio Cam Mrc5 light microscope. All fungal morphologies were quantified for length, branch rate (length µm/ number of branches) and tip numbers using ImageJ. Growth assays were repeated three times, with a minimum of 30 hyphae quantified per Dox concentration/strain.

Radial growth rates (mm) were quantified on MM agar (pH 5.6) or stress conditions. Inoculation of 10 µl volume of 1 × 10^6^ spores/ml was used, and growth rates between 72 and 120 h calculated with the indicated Dox concentration supplemented to the agar. Growth coefficients for each strain/Dox concentration were calculated thus:

$${{\frac{{{\text{Radial}}\;{\text{growth}}\;{\text{rate}}\;{\text{mutant}}\;\left( {{\text{MM}} + {\text{stress}}} \right)}}{{{\text{Radial}}\;{\text{growth}}\;{\text{rate}}\;{\text{progenitor}}\;\left( {{\text{MM}} + {\text{stress}}} \right)}}} \mathord{\left/ {\vphantom {{\frac{{{\text{Radial}}\;{\text{growth}}\;{\text{rate}}\;{\text{mutant}}\;\left( {{\text{MM}} + {\text{stress}}} \right)}}{{{\text{Radial}}\;{\text{growth}}\;{\text{rate}}\;{\text{progenitor}}\;\left( {{\text{MM}} + {\text{stress}}} \right)}}} {\frac{{{\text{Radial}}\;{\text{growth}}\;{\text{rate}}\;{\text{mutant}}\;\left( {{\text{MM}}} \right)}}{{{\text{Radial}}\;{\text{growth}}\;{\text{rate}}\;{\text{progenitor}}\;\left( {{\text{MM}}} \right)}}}}} \right. \kern-0pt} {\frac{{{\text{Radial}}\;{\text{growth}}\;{\text{rate}}\;{\text{mutant}}\;\left( {{\text{MM}}} \right)}}{{{\text{Radial}}\;{\text{growth}}\;{\text{rate}}\;{\text{progenitor}}\;\left( {{\text{MM}}} \right)}}}}.$$

#### Quantitative assessment of submerged morphology

Cultures were analysed using an Olympus szx7 stereomicroscope connected to a Canon DS126251 camera as previously described [27]. For image capture, approximately 5 ml of culture volume was poured into a 25 ml petri dish, after which morphologies were gently agitated with a pipette tip to ensure pellets were physically separated (i.e. pellets were not touching each other). For each sample, triplicate images were captured from randomly selected regions of the petri dish. Images were captured on a black background with lighting from above to illuminate fungal pellets. Triplicate replicates were conducted for each Dox/strain condition.

Fungal morphologies were quantified in ImageJ/Fiji using the morphology of dispersed and pelleted growth (MPD) plugin using default parameters [27]. Dispersed morphologies were defined as any fungal structure with an area < 500 µm^2^ and ≥ 95 µm^2^. Pellets were defined as any structure with an area ≥ 500 µm^2^. The following parameters were calculated for each fungal pellet: (i) area (µm^2^); (ii) Feret’s diameter (maximum diameter of each structure, µm); (iii) aspect ratio (maximum diameter/minimum diameter); (iv) solidity. Morphology numbers (MNs) were calculated as described earlier [16, 39]:

$${\text{Morphology}}\;{\text{number}} = \frac{{2 \times \sqrt {{\text{Area}}} \times {\text{Solidity}}}}{{\sqrt \pi \times {\text{Feret}}^{\prime}{\text{s}}\;{\text{Diameter}} \times {\text{ Aspect}}\;{\text{ratio}}}}.$$

#### Determination of fungal biomass

To determine fungal biomass, cultures were passed through filter paper, washed in sterile water, and added to pre-weighed foil envelopes. Biomass was freeze dried (minimum of 24 h) after which dry weight was determined.

#### Regression analysis

To perform the linear regression analysis, a data matrix was constructed that comprised dependent variables (secreted protein content) and independent variables (growth rate (under stress), hyphal length, tip number, pellet aspect ratio, pellet solidity, pellet diameter, and biomass dry weight. The data matrix was analysed using the Microsoft Excel ‘Data Analysis’ tool pack (Version 1808, build 10396.20023). Within Excel’s ‘Regression’ menu, default settings were used with a confidence level set at 95%. The dependent variable (*y *axis) and independent variables (*x* axis) were used as input. Independent variables were removed from the analysis if their corresponding *p*-value exceeded 0.15 in the first analysis, and 0.05 for any number of subsequent analyses. This approach allowed us to determine if, and by how much, an independent variable significantly affected the dependent variable. Furthermore, the linear regression permitted the creation of a predictive model by using the generated coefficients and intercept.

## Supplementary Information

**Additional file 1: Table S1.** Oligonucleotides used in this study. **Table S2.** A portion of the *fraA*promoter locus was DIG labelled by PCR amplification using primers 554and 784. Genomic DNA from isolate MA70.15 or putative conditional expression mutants was digested with PvuII and HindIII. The native An16g04690 locus results in a 2.3 kb fragment which acted as a control for DNA integrity. The addition of the *fraA* promoter present in the Tet-on cassette results in an additional band of predicted size indicated in Table S2. **Table S3.** Plasmids generated or used in this study. Plasmid maps will be provided on request. **Figure S1.** Southern blot confirmation of mutant isolates. Note, unannotated lanes are from mutants which either failed Southern blot testing or are not described in this study. **Figure S2.** Box plot representation of hyphal tip number following incubation on solid MM at 30 °C for 18 h. Morphogene expression was titrated using the four described Dox concentrations. Asterisks indicate where mutant isolate significantly deviates from MA70.15 control at the respective Dox concentration. Approximately 30 hyphae per strain/Dox condition were quantified. *Y*-axis: number of tips per hypha. **Figure S3.** Box plot representation of hyphal growth unit following incubation on solid MM at 30 °C for 18 h. Morphogene expression was titrated using the four described Dox concentrations. Asterisks indicate where mutant isolate significantly deviates from MA70.15 control at the respective Dox concentration. Approximately 30 hyphae per strain/Dox condition were quantified. *Y*-axis: number of tips per hyphal growth unit [hyphal length]. **Figure S4.** Pellet aspect ratio. *Y*-axis: pellet aspect ratio are represented by boxplots. + Indicates mean value, and the middle horizontal line indicates the median. Right axis: biomass is given as a percent of MA70.15 control at the respective Dox concentration. Asterisks indicate where aspect ratio of mutant isolate significantly deviates from MA70.15 control at the respective Dox concentration. Values are from triplicate biological replicates. **Figure S5.** Pellet solidity. *Y*-axis: pellet solidity are represented by boxplots. + Indicates mean value, and the middle horizontal line indicates the median. Right axis: biomass is given as a percent of MA70.15 control at the respective Dox concentration. Asterisks indicate where solidity of mutant isolate significantly deviates from MA70.15 control at the respective Dox concentration. Values are from triplicate biological replicates.

## Data Availability

The data sets used and/or analysed during the current study are available from the corresponding author on reasonable request.
