# Supplementary material for: Regression modelling of conditional morphogene expression links and quantifies the impact of growth rate, fitness and macromorphology with protein secretion in Aspergillus niger
Source: Biotechnol Biofuels Bioprod. 2023 Jun 2;16:95. doi: 10.1186/s13068-023-02345-9 (PMC10239186; doi:10.1186/s13068-023-02345-9)
Supplement: Supplementary file 1 — Additional file 1: Table S1. Oligonucleotides used in this study. Table S2. A portion of the fraApromoter locus was DIG labelled by PCR amplification using primers 554and 784. Genomic DNA from isolate MA70.15 or putative conditional expression mutants was digested with PvuII and HindIII. The native An16g04690 locus results in a 2.3 kb fragment which acted as a control for DNA integrity. The addition of the fraA promoter present in the Tet-on cassette results in an additional band of predicted size indicated in Table S2. Table S3. Plasmids generated or used in this study. Plasmid maps will be provided on request. Figure S1. Southern blot confirmation of mutant isolates. Note, unannotated lanes are from mutants which either failed Southern blot testing or are not described in this study. Figure S2. Box plot representation of hyphal tip number following incubation on solid MM at 30 °C for 18 h. Morphogene expression was titrated using the four described Dox concentrations. Asterisks indicate where mutant isolate significantly deviates from MA70.15 control at the respective Dox concentration. Approximately 30 hyphae per strain/Dox condition were quantified. Y-axis: number of tips per hypha. Figure S3. Box plot representation of hyphal growth unit following incubation on solid MM at 30 °C for 18 h. Morphogene expression was titrated using the four described Dox concentrations. Asterisks indicate where mutant isolate significantly deviates from MA70.15 control at the respective Dox concentration. Approximately 30 hyphae per strain/Dox condition were quantified. Y-axis: number of tips per hyphal growth unit [hyphal length]. Figure S4. Pellet aspect ratio. Y-axis: pellet aspect ratio are represented by boxplots. + Indicates mean value, and the middle horizontal line indicates the median. Right axis: biomass is given as a percent of MA70.15 control at the respective Dox concentration. Asterisks indicate where aspect ratio of mutant isolate significantly deviates from MA70.15 [file 13068_2023_2345_MOESM1_ESM.docx]

**Regression modelling of conditional morphogene expression links and quantifies the impact of growth rate, fitness and macromorphology with protein secretion in *Aspergillus niger***

Cairns, T. C.^1^*§, de Kanter, T. ^1^*, Zheng, X. Z.^2,3,4,5^, Zheng, P.^2,3,4,5^, Sun, J.^2.3,4,5^, Meyer. V^1^.§

*Authors contributed equally

§ Corresponding authors

^1^Technische Universität Berlin, Institute of Biotechnology, Chair of Applied and Molecular Microbiology,

Straße des 17. Juni 135, 10623 Berlin, Germany

^2^ Tianjin Institute of Industrial Biotechnology, Chinese Academy of Sciences, Tianjin, 300308, People’s Republic of China

^3^ Key Laboratory of Systems Microbial Biotechnology, Chinese Academy of Sciences, Tianjin 300308, People’s Republic of China

^4^ University of Chinese Academy of Sciences, Beijing, 100049 China

^5^ College of Biotechnology, Tianjin University of Science & Technology, Tianjin, 300457 China

Timothy C. Cairns: [t.cairns@tu-berlin.de](mailto:t.cairns@tu-berlin.de) ORCID: 0000-0001-7106-224X

Tom de Kanter: [tomdekanter@gmail.com](mailto:tomdekanter@gmail.com) ORCID: 0000-0001-8058-906X

Xiaomei Zheng: [zheng_xm@tib.cas.cn](mailto:zheng_xm@tib.cas.cn) ORCID: 0000-0001-9136-0666

Ping Zheng: [zheng_p@tib.cas.cn](mailto:zheng_p@tib.cas.cn): ORCID: 0000-0001-9434-9892

Jibin Sun: [sun_jb@tib.cas.cn](mailto:sun_jb@tib.cas.cn): ORCID: 0000-0002-0208-504X

Vera Meyer: [vera.meyer@tu-berlin.de](mailto:vera.meyer@tu-berlin.de), ORCID 0000-0002-2298-2258

**Contact details for corresponding authors:**

Timothy C. Cairns, Tel.: +49 30 314 72750, Fax: +49 30 314 72922, E-mail: [t.cairns@tu-berlin.de](mailto:t.cairns@tu-berlin.de)

Vera Meyer, Tel.: +49 30 314 72750, Fax: +49 30 314 72922, E-mail: [vera.meyer@tu-berlin.de](mailto:vera.meyer@tu-berlin.de)

**Supplemental Table S1- Oligonucleotides used in this study**

| Name |  | Sequences 5′ 🡪 3′ | |
| --- | --- | --- | --- |
| sgRNAs |  |  |  |
| 21An02g09230A12F |  | caccATAAGGCGACTCGGCATTTA |  |
| 21An02g09230A12R |  | aaacTAAATGCCGAGTCGCCTTAT |  |
| 23An08g04400A59F |  | caccCTCTCTAGACAGTCATTATG |  |
| 23An08g04400A59R |  | aaacCATAATGACTGTCTAGAGAG |  |
| 26_sg_An01g13700_Fw |  | caccAGGGTCTATATCGTACTTGA |  |
| 26_sg_An01g13700_rev |  | aaacTCAAGTACGATATAGACCCT |  |
| 28_sg_An12g10750_Fw |  | caccTCCCAGGAGCCGCCAGAGTT |  |
| 28_sg_An12g10750_rev |  | aaacAACTCTGGCGGCTCCTGGGA |  |
| 36_sg_An08g04960_Fw |  | caccGCGGTCGCATATTCCGTCGA |  |
| 36_sg_An08g04960_rev |  | aaacTCGACGGAATATGCGACCGC |  |
| 38_sg_An11g02670_Fw |  | caccCGCGGAGCGATACACTCAAT |  |
| 38_sg_An11g02670_rev |  | aaacATTGAGTGTATCGCTCCGCG |  |
| 39_sg_An16g01520_Fw |  | caccACTTCGTGAGCTACGCTGCT |  |
| 39_sg_An16g01520_rev |  | aaacAGCAGCGTAGCTCACGAAGT |  |
| 51_sg_An07g01060_Fw |  | caccACACGCGATCTACCGTGGAA |  |
| 51_sg_An07g01060_rev |  | aaacTTCCACGGTAGATCGCGTGT |  |
| 53_sg_An08g10160_Fw |  | caccGATGCCGAGCGGCATAACTA |  |
| 53_sg_An08g10160_rev |  | aaacTAGTTATGCCGCTCGGCATC |  |
|  |  |  |  |
| sgRNA amplification |  |  |  |
| M13 forward |  | GTAAAACGACGGCCAGTG |  |
| M13 Reverse |  | CAGGAAACAGCTATGAC |  |
|  |  |  |  |
| Donor DNA amplification |  |  |  |
| MH_An02g09230A12_ptrpc_F |  | ataaatgtgaccgtctctcgagaactgaacctcaatatcgGACGTTAACTGATATTGAAG |  |
| MH_An02g09230A12_pmin_R |  | tcgtcggggacggacgccgtaatataaggcgactcggCATGGTGTTTAAACGGTGATGTC |  |
| MHr_An08g04400A60_ptrpc_F |  | gtcgattttattatgattgttaagtcatttgaactgttttGACGTTAACTGATATTGAAG |  |
| MH_An08g04400A19_pmin_R |  | gggtgcgcggaggaatagggatagggagattggatgcCATGGTGTTTAAACGGTGATGTC |  |
| 26_MH_An01g13700_ptrypC |  | ctctgagcagatgttgtgcacttctcttagaagttgtgagGACGTTAACTGATATTGAAG |  |
| 26_MH_An01g13700_pmin_R |  | gacacctgatggagcccacatctatcgcagcatcagtcatGGTGTTTAAACGGTGATGTC |  |
| 28_MH_An12g10750_ptrypC |  | caagttggaatcgagaagctgatgacagaccaatcttcttGACGTTAACTGATATTGAAG |  |
| 28_MH_An12g10750_pmin_R |  | ctcggccaccagggcgctgggctgaaggttcaccattcatGGTGTTTAAACGGTGATGTC |  |
| 36_MH_An08g04960_ptrypC |  | ctcccccgaccgctgggaatccctctcctgccgtcgatcaGACGTTAACTGATATTGAAG |  |
| 36_MH_An08g04960_pmin_R |  | cgatcgggtcgaaaaggggttgatcaagcgctgccgccatGGTGTTTAAACGGTGATGTC |  |
| 38_MH_An11g02670_ptrypC |  | ttccagggcgccctacagaaccggttatccccttcgaaggGACGTTAACTGATATTGAAG |  |
| 38_MH_An11g02670_pmin_R |  | acttacctcggagggacgtgcggggggtttgggaagacatGGTGTTTAAACGGTGATGTC |  |
| 39_MH_An16g01520_ptrypC |  | ccattgggaacagcgcgcttcatcgatcctccttccggcgGACGTTAACTGATATTGAAG |  |
| 39_MH_An16g01520_pmin_R |  | catcgcccccagggccgacgtaccctccgcccccagccatGGTGTTTAAACGGTGATGTC |  |
| 51_MH_An07g01060_ptrypC |  | gtcgcatcttacatctggttgcgacattagacgcatcatcGACGTTAACTGATATTGAAG |  |
| 51_MH_An07g01060_pmin_R |  | aggcaaggaagggcaatgctgcaagagttaggaagtgcatGGTGTTTAAACGGTGATGTC |  |
| 53_MH_An08g10160_ptrypC |  | cccagctatcatcattcatcccatacctGACGTTAACTGATATTGAAG |  |
| 53_MH_An08g10160_pmin_R |  | aagtcttgccatctgaagaaggatctctcttagccaacatGGTGTTTAAACGGTGATGTC |  |
|  |  |  |  |
| Tet-on gDNA verification |  |  |  |
| An02g09230-V-F |  | CATGGACTGTAACCCCTGCT |  |
| An02g09230-V-R |  | GCCCTTGAAAGCTTCGTGTA |  |
| An08g04400-V-F |  | GGTGGATGGATCAAGTCTGG |  |
| An08g04400-V-R |  | GATTGGCATCACTGGGATCT |  |
| 26_Ver_An01g13700_F |  | TCCACGCCTGGATGAGAAAC |  |
| 26_Ver_An01g13700_R |  | TGGACAAGATCGACAGTGCC |  |
| 28_Ver_An12g10750_F |  | GCACTGTGTACTGGAGACGG |  |
| 28_Ver_An12g10750_R |  | GTTCGCAACCGGAAAGATGG |  |
| 36_Ver_An08g04960_F |  | GCTTGCTTGACCTGACTTGC |  |
| 36_Ver_An08g04960_R |  | GGCCTTCCGGCTTGATCTTA |  |
| 38_Ver_An11g02670_F |  | CCAGACAAACATGACCGGGA |  |
| 38_Ver_An11g02670_R |  | GTTCCCATTTTACGCCCGTG |  |
| 39_Ver_An16g01520_F |  | TGGCGGTTTAGACGGAGTTT |  |
| 39_Ver_An16g01520_R |  | GGGGTGTGACGAAGGAATGA |  |
| 51_Ver_An07g01060_F |  | CTCGCAAGCTACTCCCGAAT |  |
| 51_Ver_An07g01060_R |  | CACCCCTTCTTCCACAGCAT |  |
| 53_Ver_An08g10160_F |  | GTTCTGGAAACAACGACGGC |  |
| 53_Ver_An08g10160_R |  | TGATGCGTGTAAGCGGGAAT |  |

**Supplemental Table S2: Predicted sizes of Southern blot probe hybridization using the DIG labelled *fraA* probe**

A portion of the *fraA* (An16g04690) promoter locus was DIG labelled by PCR amplification using primers 554 (ccctcggctggtctgtctta) and 784 (tttggcggtttgttgctggc). Genomic DNA from isolate MA70.15 or putative conditional expression mutants was digested with PvuII and HindIII. The native An16g04690 locus results in a 2.3 kb fragment which acted as a control for DNA integrity. The addition of the *fraA* promoter present in the Tet-on cassette results in an addition band of predicted size as indicated.

| **Strain** | **Predicted hybridisation size of native *fraA* promoter** | **Predicted hybridisation size following integration of Tet-on at the target locus** |
| --- | --- | --- |
| **MA70.15** | 2.3 kb | NONE |
| **TK26.2** | 2.3 kb | 7.1 kb |
| **TK28.5** | 2.3 kb | 8.5 kb |
| **TK38.5** | 2.3 kb | 7.7 kb |
| **TK39.1** | 2.3 kb | 5.1 kb |
| **TK51.1** | 2.3 kb | 5.6 kb |
| **TK53.1** | 2.3 kb | 7.1 kb |

**Supplemental table S3- Plasmids generated or used in this study. Plasmid maps will be provided on request.**

| Plasmid | Relevant properties | References |
| --- | --- | --- |
| pTK18.1 | sgRNA template for gene 21 | This study |
| pTK19.1 | sgRNA template for gene 23 | This study |
| pTK4.1 | sgRNA template for gene 26 | This study |
| pTK1.1 | sgRNA template for gene 28 | This study |
| pTK2.1 | sgRNA template for gene 36 | This study |
| pTK3.1 | sgRNA template for gene 38 | This study |
| pTK20.1 | sgRNA template for gene 39 | This study |
| pTK23.1 | sgRNA template for gene 51 | This study |
| pTK24.1 | sgRNA template for gene 53 | This study |
| pXM7 | Vector for sgRNA template integration and ampicillin resistance | Zheng *et al.*, 2018 |
| pTC1.13 | Contains the Tet-on cassette and hygromycin resistance | Cairns *et al.*, 2022 |
| pCas9 | Cas9 enzyme | Zheng *et al.*, 2018 |

**
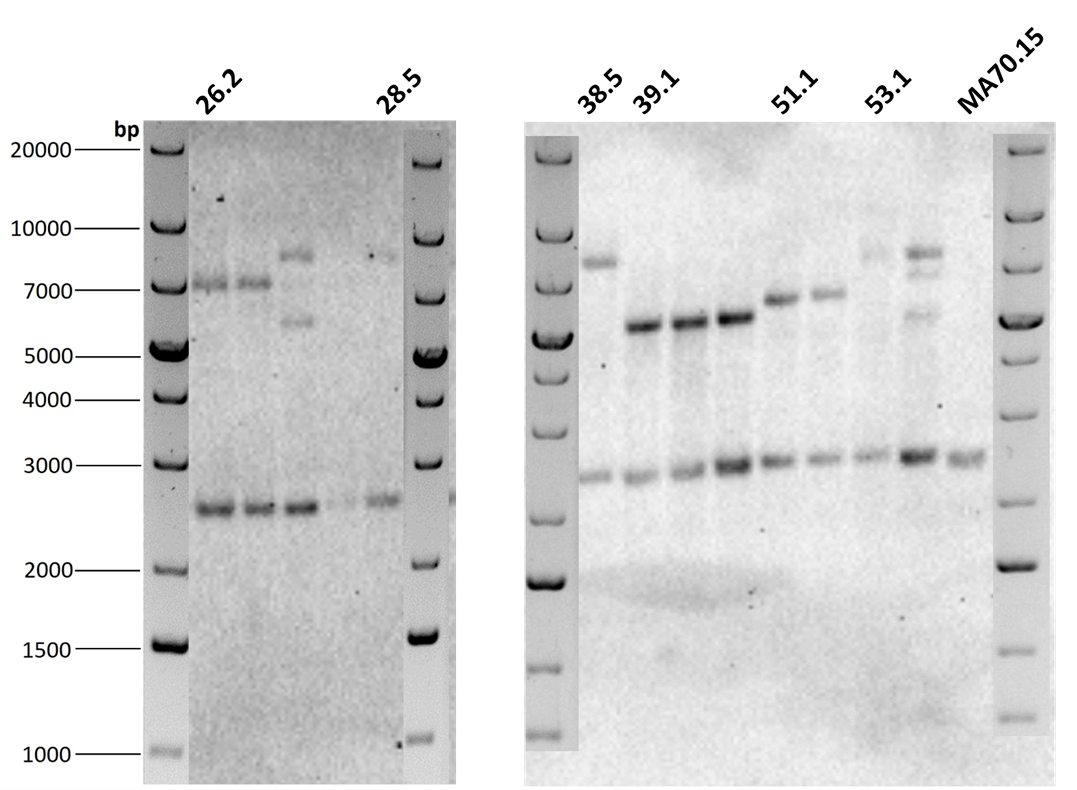
**

**Supplemental Figure S1: Southern blot confirmation of mutant isolates.** Note, unannotated lanes are from mutants which either failed Southern blot testing or are not described in this study.

**
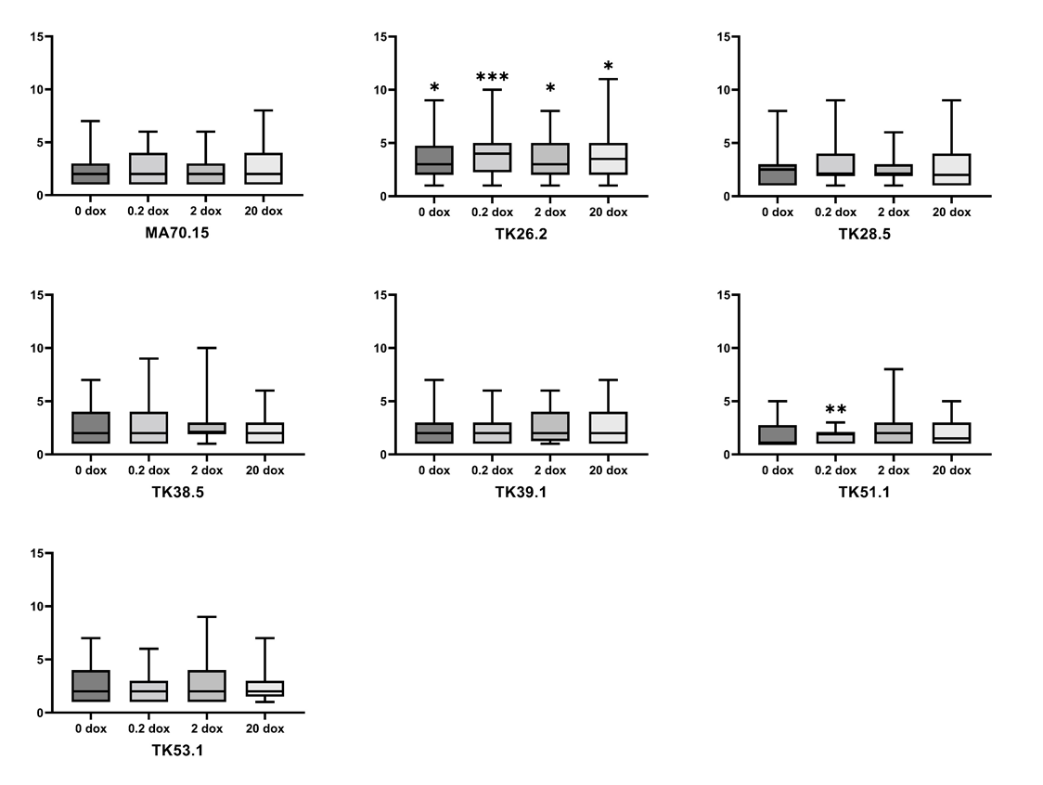
**

**Supplemental Figure S2: Box plot representation of hyphal tip number following incubation on solid MM at 30°C for 18 hours.** Morphogene expression was titrated using the four described Dox concentrations. Asterisks indicate where mutant isolate significantly deviates from MA70.15 control at the respective Dox concentration (t-test). Approximately 30 hyphae per strain/Dox condition were quantified. Y-axis: number of tips per hypha.

**
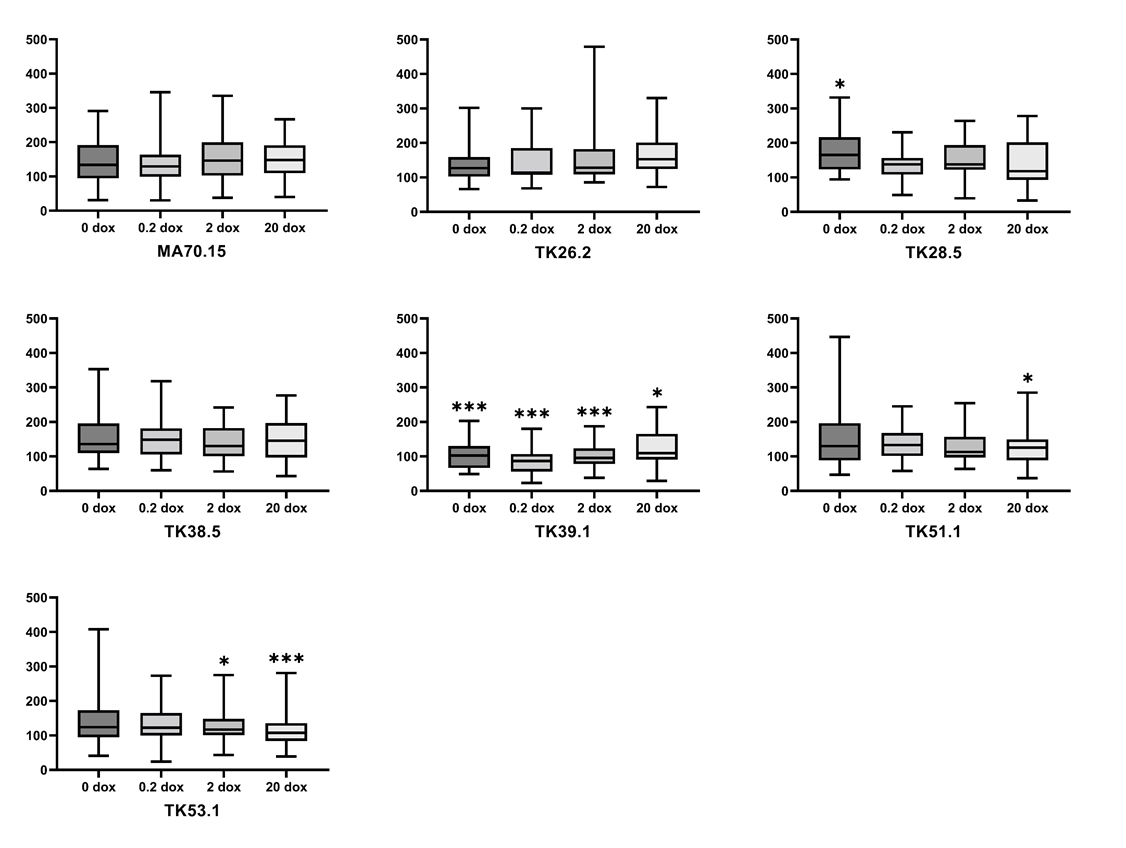
**

**Supplemental Figure S3: Box plot representation of hyphal growth unit following incubation on solid MM at 30°C for 18 hours.** Morphogene expression was titrated using the four described Dox concentrations. Asterisks indicate where mutant isolate significantly deviates from MA70.15 control at the respective Dox concentration (t-test). Approximately 30 hyphae per strain/Dox condition were quantified. Y-axis: number of tips per hyphal growth unit (hyphal length (µm/tip number).

**
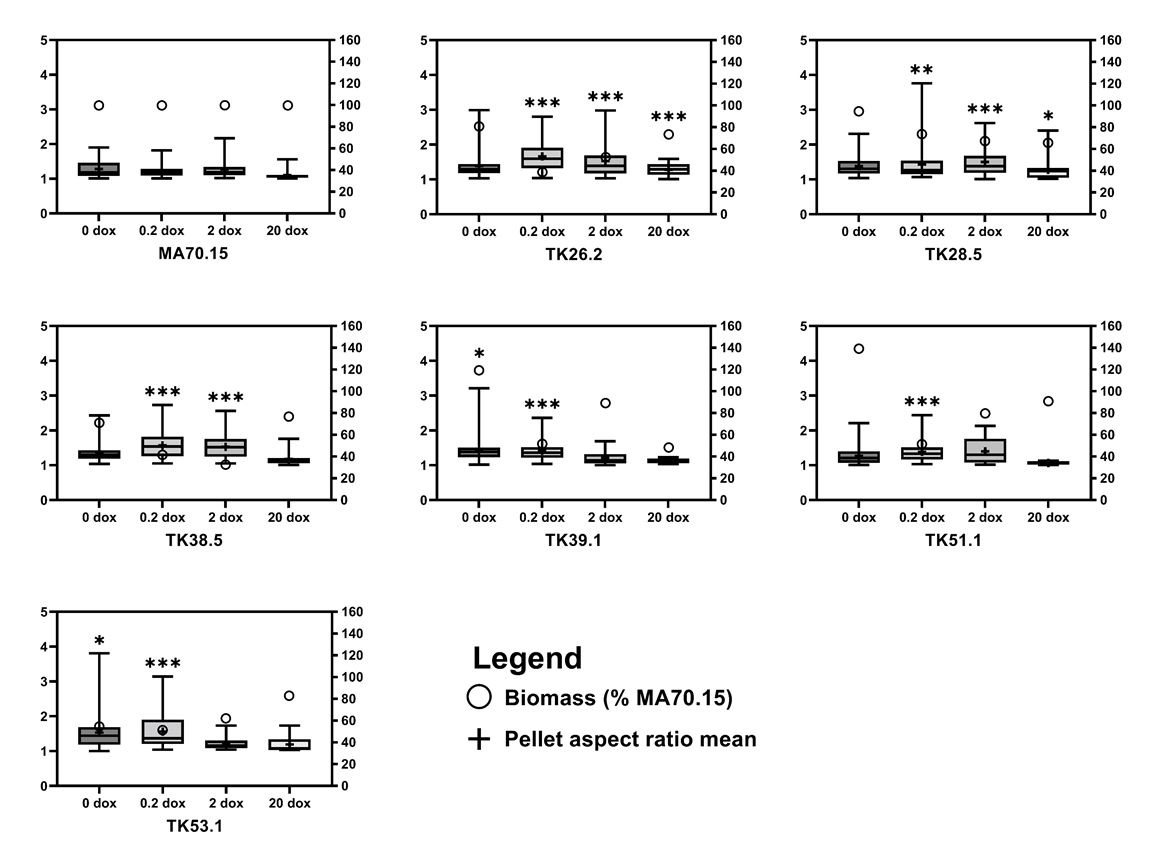
**

**Supplemental Figure S4: Pellet aspect ratio.** Y-axis: pellet aspect ratio are represented by boxplots. + indicates mean value, and the middle horizontal line indicates the median. Right axis: Biomass is given as a percent of MA70.15 control at the respective Dox concentration. Asterisks indicate where aspect ratio of mutant isolate significantly deviates from MA70.15 control at the respective Dox concentration (t-test). Values are from triplicate biological replicates.

**
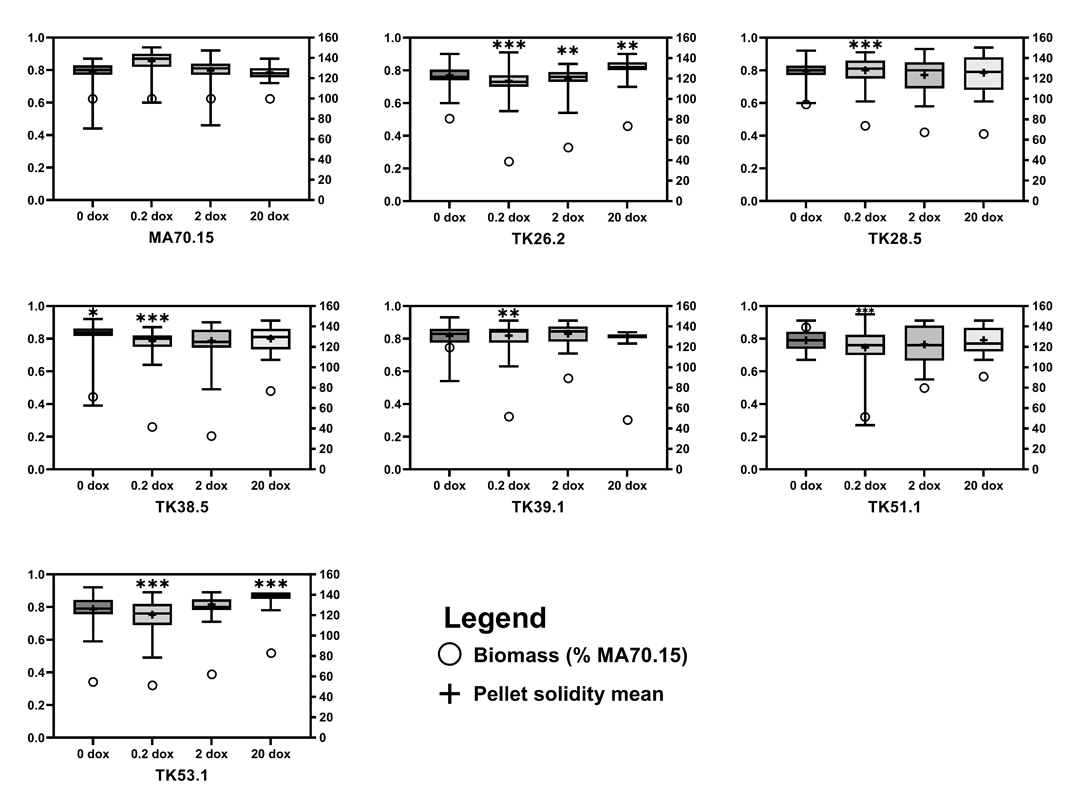
**

**Supplemental Figure S5: Pellet solidity.** Y-axis: pellet solidity are represented by boxplots. + indicates mean value, and the middle horizontal line indicates the median. Right axis: Biomass is given as a percent of MA70.15 control at the respective Dox concentration. Asterisks indicate where solidity of mutant isolate significantly deviates from MA70.15 control at the respective Dox concentration (t-test). Values are from triplicate biological replicates.
